# RNA sequencing provides evidence for functional variability between naturally co-existing *Alteromonas macleodii* lineages

**DOI:** 10.1186/1471-2164-15-938

**Published:** 2014-10-26

**Authors:** Nikole E Kimes, Mario López-Pérez, Eva Ausó, Rohit Ghai, Francisco Rodriguez-Valera

**Affiliations:** Evolutionary Genomics Group, División de Microbiología, Universidad Miguel Hernández, Apartado 18, San Juan, Alicante, 03550 Spain; Department of Medicine, University of California San Francisco, 513 Parnassus Ave, San Francisco, CA 94143 USA

**Keywords:** *Alteromonas macleodii*, Genomic diversity, RNA-seq, Transcriptomics, Genomic islands, CRISPR

## Abstract

**Background:**

*Alteromonas macleodii* is a ubiquitous gammaproteobacterium shown to play a biogeochemical role in marine environments. Two *A. macleodii* strains (AltDE and AltDE1) isolated from the same sample (i.e., the same place at the same time) show considerable genomic differences. In this study, we investigate the transcriptional response of these two strains to varying growth conditions in order to investigate differences in their ability to adapt to varying environmental parameters.

**Results:**

RNA sequencing revealed transcriptional changes between all growth conditions examined (e.g., temperature and medium) as well as differences between the two *A. macleodii* strains within a given condition. The main inter-strain differences were more marked in the adaptation to grow on minimal medium with glucose and, even more so, under starvation. These differences suggested that AltDE1 may have an advantage over AltDE when glucose is the major carbon source, and co-culture experiments confirmed this advantage. Additional differences were observed between the two strains in the expression of ncRNAs and phage-related genes, as well as motility.

**Conclusions:**

This study shows that the genomic diversity observed in closely related strains of *A. macleodii* from a single environment result in different transcriptional responses to changing environmental parameters. This data provides additional support for the idea that greater diversity at the strain level of a microbial community could enhance the community’s ability to adapt to environmental shifts.

**Electronic supplementary material:**

The online version of this article (doi:10.1186/1471-2164-15-938) contains supplementary material, which is available to authorized users.

## Background

*Alteromonas macleodii,* a marine gammaproteobacterium*,* is distributed globally and especially abundant in the temperate and tropical latitudes [1–4]. A quintessential marine *r*-strategist, it plays an important biogeochemical role in marine waters, and recent metatranscriptomic studies have highlighted its important role in marine dissolved organic carbon and nitrogen recycling [5, 6]. Isolations and genome sequencing of a number of *A. macleodii* strains from different regions of the world have also contributed towards an increasing understanding of this microbe’s ecological niche [1, 2]. It has been known for years that *Alteromonas* cells are associated to the particulate fraction [7] and capable of nutrient cycling [8]. More recently, genome sequencing provided evidence for further specialization within *A. macleodii* based on the particulate matter associated with each strain, suggesting that most surface isolates are associated to small, slow sinking particles, while deeper isolates live on larger faster sinking aggregates [9]. The two strains studied here, AltDE and AltDE1, were isolated from a single deep (1000 m) seawater sample from the South Adriatic (Mediterranean Sea) and both belong to the “large particle” or “deep” ecotype or subspecies [3]. These two strains have a high average nucleotide identity (ANI, 98.51%) when comparing their core genomes (3631 shared genes, ~80% of genome) [3]. However, these concurrent isolates still differ from each other in 9 flexible genomic islands, which are located in the same genomic context yet contain different genes plus a number of small insertions and deletions, some involving complete gene clusters [3]. The collective pool of genes detected among the strains in a single species is referred to as the pan-genome [10, 11] and has now been described in many microorganisms [10–15]. However, most of these studies examine the gene pool of strains that are not found together in the same natural population. This leads researchers to assign their differences to specific adaptations to their habitat. In the case of AltDE and AltDE1, isolated from the same sample, the considerable genomic variation observed has been interpreted as contributing to adaptation to even more specialized microniche partitioning [3]. In fact, the coexistence of such closely related, yet distinct, *A. macleodii* strains within a single sample brings up a number of questions. For example, do the flexible genomic regions represent functional differences between the strains under ecologically relevant conditions? It has been speculated that the gene diversity presented by pan-genomes provides a broader range of ecological capabilities and adaptability within a species [16], However, more evidence to support such assertions is required. These naturally coexisting strains of *A. macleodii* provide an excellent opportunity to examine the responses of each strain, including the transcriptional expression of both the core and flexible genome, under different conditions in an attempt to identify the nature of adaptive advantages conferred by their genomic differences.

We hypothesized that the two strains would exhibit distinct transcriptional responses to a variety of environmental conditions. Thus providing evidence for the differences expected from the variable flexible gene pools. We also wanted to examine whether or not their genomic differences affect their competitiveness in co-cultures under ecologically relevant conditions, and whether their transcriptional profiles provide clues to the observed responses. To investigate these hypotheses, we performed RNA-sequencing on AltDE and AltDE1 grown in four different conditions, providing a total of eight transcriptomes. The four conditions included growth in rich medium at two different temperatures (13°C and 25°C) and minimal medium at 25°C with glucose as the sole carbon source and subsequently two days after removal of the carbon source (i.e., starvation). Furthermore, we examined the population dynamics of the two strains during co-culture to assess the effect of growth conditions on the population structure. Our results show that AltDE and AltDE1 exhibit specific adaptations, giving each strain an advantage under different ecological conditions in large part due to expression of their flexible genomes. This supports the theory that genetic diversity within a population provides a broader arsenal for adaptation within a given community.

## Results and discussion

### Overview of RNA-seq results

In this study we used different growth conditions to explore the differences between two distinct, yet closely related, *A. macleodii* strains isolated from the same environmental sample: AltDE and AltDE1. The conditions included two temperatures (13 and 25°C), which represent normal temperature extremes in the Mediterranean (winter and summer respectively), and two growth media, a commonly used laboratory medium (marine broth or nutrient-rich medium, RM), used at both temperatures and a minimal medium with glucose (MMG), only at 25°C. Another condition assayed was starvation (STR), in which cells grown in MMG at 25°C were deprived of any carbon source for 48 hours. Using these growth conditions (N = 4 for each strain), we sequenced the cDNA of AltDE and AltDE1, resulting in over 50 million paired end reads that we were able to map to the AltDE and AltDE1 genomes accordingly (Table 1).

**Table 1:** **RNA sequencing results**

Strain	AltDE				AltDE1			
Growth Conditions	RM ^a^-13°C	RM ^a^-25°C	MMG ^b^-25°C	STR ^c^-25°C	RM ^a^-13°C	RM ^a^-25°C	MMG ^b^-25°C	STR ^c^-25°C
Total number of reads	3,444,176	11,503,336	8,986,648	2,995,620	6,230,679	7,840,241	13,514,524	14,695,977
Reads mapped to ribosomal operons	3,206,468	10,852,030	8,764,784	2,940,326	5,812,308	7,342,658	13,314,002	14,668,090
Percentage of reads mapped to ribosomal operons	93.1	94.3	97.5	98.2	93.2	93.7	98.5	99.8
Chromosome length (nt)	4,480,937				4,643,844			
Average coverage per nucleotide^d^	8.7X	14.8X	5.0X	1.2X	8.9X	10.5X	4.1X	0.6X
Percentage of CDS expressed	76.9	90.3	86.5	86.2	82.7	88.8	87.6	43.7
Reads mapped in the chromosome	387,708	661,306	221,864	55,294	412,700	487,487	191,820	27,243
Reads mapped to CDS	258,240	454,504	160,098	40,566	275,125	325,206	139,930	21,824
Percentage of reads mapped to IR	33.4	30.2	27.8	26.6	33.3	33.3	27.1	19.8
Percentage of reads mapped to ribosomal proteins^e^	21	22.9	11.2	6	20.9	20.1	4	1.2
Plasmid length (nt)	--	--	--	--	303,282			
Average coverage per nucleotide	--	--	--	--	1.9X	3.3X	2.9X	0.2X
Reads mapped to plasmid	--	--	--	--	5,671	10,096	8,702	644

CDS, coding DNA sequence; IR, intergenic region.

^a^nutrient rich marin medium.

^b^minimal medium with 1% glucose.

^c^minimal medium with 1% glucose followed by two days of minimal medium without glucose.

^d^number of mapped reads X 100/number of nucleotides in the chromosome.

^e^percentage of CDS.

We have sequenced total RNA (both rRNA and mRNA) from *A. macleodii* transcriptomes to reduce bias introduced by mRNA enrichment [17]. The depth of sequencing that can now be achieved from Illumina sequencing is sufficient to provide adequate coverage of the mRNA without rRNA depletion [18]. As expected, the vast majority of reads (ranging from 93.1 to 99.8%) in all eight transcriptomes mapped to the rRNA operons (Table 1). For the growing cells, the two nutrient rich conditions revealed lower percentages of rRNA transcripts (93.1 – 94.3%) than the minimal medium (97.5 - 98.5%). There might be a larger ribosome concentration in the minimal medium grown cells or some specific genes are much more expressed in the rich medium (see below). The percentage of CDS expressed was similar in both growth media at 25°C (ca. 90%). However, it was significantly smaller at 13°C (76.9 and 82.7% in DE and DE1 respectively), which might be a consequence of the lower growth rate at this temperature. Even after removal of the rRNA reads, we obtained sensible genome coverage ranging from 4.1X to 14.8X. In the starvation (STR) experiments, the two strains differed markedly with much larger amounts of rRNA reads in the case of AltDE1. Where the AltDE genome remained largely active at the transcriptional level under starvation, AltDE1 appeared to have shut down transcription through most of its genome. It has been reported that under certain stress conditions, such as starvation, bacteria respond to adverse conditions reducing translational activity, leading to slow cell growth [19].

**Figure 1:**
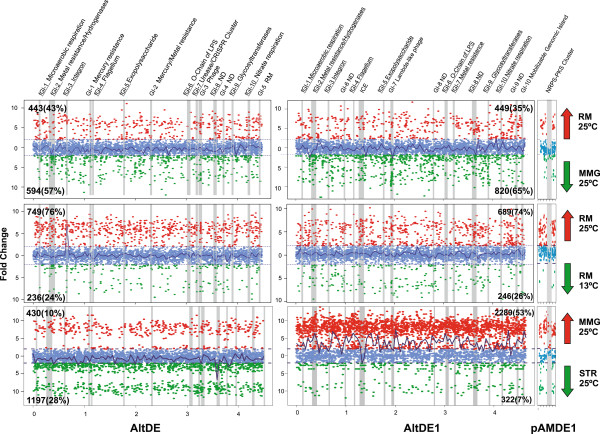
**Fold changes across the genomes of AltDE and AltDE1.** Genomic islands (GI) and flexible genomic islands (fGI) are plotted in grey bars in both genomes. The temperature plots on the left are comparisons at two different temperatures (25°C and 13°C). The growth medium plots on the right are the comparisons between rich medium (RM) vs minimal medium with glucose (MMG) at 25°C. In each plot the genes that have a fold change of >2 or < −2 are colored red and green respectively. In the temperature panels, red represents up regulation at 25°C, while green represents up regulation at 13°C. In the growth medium panels red represents up regulation in RM, while green represents up regulation in MMG. Genes in blue are those that do not have a fold change of >2/<−2, and are delineated by dashed horizontal lines. A lowness fit of the expression values is shown along the genome. The numbers of these genes and the percentage of total genes affected are shown in bold in the corner of each plot. Brief descriptions of the islands: fGI-1, Microaerobic respiration; fGI-2, Metal resistance/Hydrogenases; fGI-3, ND; fGI-4, Flagellum; fGI-5, Exopolysaccharide; fGI-6, O-chain of LPS; fGI-7, Urease/CRISPR cluster; fGI-8, ND; fGI-9, Glycosyltransferases; GI-1, Mercury resistance; GI-2, Mercury/Metal resistance; GI-3, Phage; GI-4, ND; GI-5, Nitrate respiration; GI-6, ND; GI-7, Lambda-like phage; GI-8, ND; GI-9, ND; GI-10, Mobilizable Genomic Island (MGI). ND, not defined.

We identified differentially expressed genes between the conditions analyzed using a cut-off criteria of ≥2 fold log increases for up-regulated genes (Figure 1). A general up-regulation of genes across the AltDE and AltDE1 genomes at 25°C (over 75% of the total differentially expressed) compared to 13° was detected (Figure 1). Although AltDE and AltDE1 exhibited similar transcriptional fingerprints for the most part at 25°C, there was a large peak of up-regulation observed that was unique to AltDE (Figure 1). Examination of these genes revealed the up-regulation of a flexible region (fGI-3) that consists entirely of an integron [20] in both strains. Despite the fact that the integron integrases were 99.1% identical in both strains, the gene cassettes inserted in the downstream region were totally different. Cassettes are promoterless and conditioned to the Integron *Pc* promoter that is embedded in the integrase [21]. However, in the case of the *A. macleodii* strains studied here each cassette displayed independent regulation. For example, all the toxin-antitoxin systems (suggested to be involved in the stabilizing of the integron) were up-regulated in STR, while the nearest gene cassette to the integron integrase in AltDE1 showed no expression under any condition.

**Figure 2:**
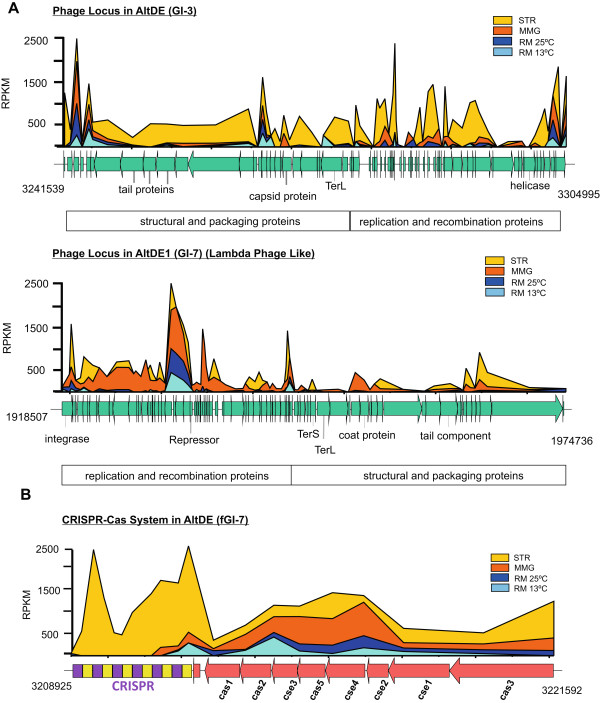
**CRISPR/Cas system and Prophages expression. A**. Expression of phage loci in the AltDE (GI-3) and AltDE1 (GI-7) genomes. **B**. Expression of CRISPR-Cas locus in the AltDE genome (fGI-7). Gene expression data (expressed as RPKM) is shown mapped to the genes (MMG: minimal medium with glucose, STR: starvation, minimal medium without glucose, RM: Rich Medium).

Broad scale comparison also shows that the two strains differ in their response to the growth medium, with AltDE1 overexpressing many more genes in MMG than AltDE (Figure 1). Also in AltDE1, we observed six unique peaks of up-regulation. Three of them in RM correspond to regions encoding ribosomal proteins. In contrast, two peaks in the MMG were associated with pilus and antioxidant production, while the third peak corresponded to a genomic island (GI-7) encoding a different lambda-like phage in each strain. Interestingly, in AltDE1 GI-7 is expressed in both MMG and STR, while the AltDE phage-like region (GI-3) is only expressed under the most severe starving condition (Figure 2). In this comparison, for example we identified a considerable change in relative expression of a number of genes which are involved in nitrogen metabolism (Additional file 1: Table S1). Among these, a gene coding for a PII-like signal transduction protein (GlnK) that regulates the expression of *glnA-ntrBC* operon [22] and an ammonium transporter (*amtB*) during nitrogen starvation showed the greatest expression in MMG in comparison to RM (11.02 and 13.97 fold change in DE and DE1 respectively) in both strains. Likewise, the *glnA* gene, which encodes glutamine synthetase that is the central enzyme for nitrogen assimilation from ammonia into glutamine and the nitrate ABC transporter (*ntrC*), were also up-regulated. The same pattern was conserved for most of the genes involved in ammonium uptake and assimilation as well as genes coding for nitrogen assimilatory enzymes, e.g., *nirBD.* Although all of these nitrogen related genes were induced in both strains in MMG, most of these genes showed stronger induction levels for AltDE1 than AltDE (Additional file 1: Table S1).

The variability between AltDE and AltDE1 appeared to be greater in the differentially expressed genes between MMG and STR compared to the variability observed between the other conditions (Figure 1). Despite the low proportion of genes that were expressed in AltDE1 STR, 53% were differentially expressed compared to MMG. The genes of the Integrative and Conjugative Element (ICE) which were expressed at higher levels under STR, represented the only significant difference between these two conditions in AltDE1. AltDE exhibited the reverse situation suggesting that this strain may have an advantage under circumstances of nutrient deprivation. We also identified a marked peak at STR in AltDE corresponding to the fGI-8, but no function could be inferred for this GI (Figure 1). Due to the high similarity between the two strains (3631 core genes), these results highlight the relative importance of the flexible genome in the different adaptive responses observed of the species especially during STR conditions.

AltDE1 also carries a 300-kb conjugative plasmid (pAMDE1) previously described by Gonzaga et al. [3]. The expression profile of the hybrid non-ribosomal peptide synthetase-polyketide synthase (NRPS-PKS) gene cluster, which was predicted to produce a bleomycin-like compound and contains a bleomycin-resistance gene [23], revealed increased expression in MMG compared to RM or STR (Additional file 2: Figure S1). This suggests that glucose is an inductor for the production of this compound.

**Figure 3:**
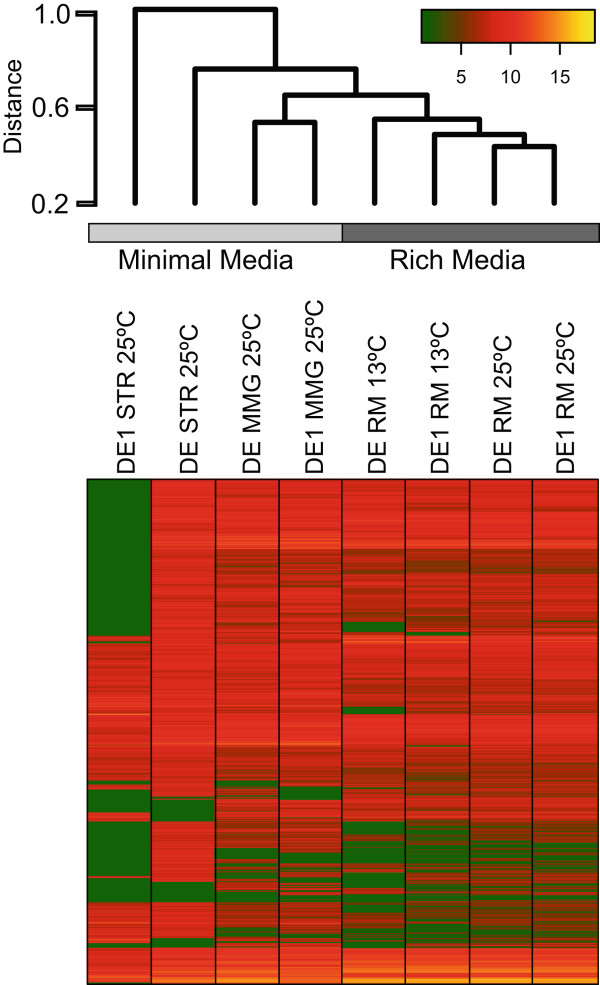
**Hierarchical clustering of expression data for the orthologous genes in AltDE and AltDE1 in all conditions examined.** A color key is shown on top right. Data shown are log2 transformed RPKM values. MMG: minimal medium with glucose, STR: starvation, minimal medium without glucose, RM: Rich Medium.

We also performed hierarchical clustering (Figure 3) using the expression of orthologous genes in AltDE and AltDE1 (i.e., the core genome) and revealed that the main driver of differential gene expression was the growth medium followed by temperature and finally the strain. This indicates that both strains’ core genomes (3631 genes) react similarly to the variations in growth conditions. Furthermore, the fact that the core genome contributes to the majority (67-79%) of differentially expressed genes in each condition (Table 2) clearly demonstrates its role in the observed changes. These core genes include regulatory elements (e.g. ncRNAs and transcription factors) and nutrient cycling genes (e.g. carbon and nitrogen cycling genes).

**Table 2:** **Number of genes differentially expressed in AltDE and AltDE1**

Strain	AltDE			AltDE1		
**Total genes in the genome**	4389			4748		
**Total core genes in the genome (%)** ^**a**^	3631 (83)			3631 (76)		
**Total flexible genes in the genome (%)** ^**a**^	758 (17)			1117 (24)		
**Comparison**	**Temperature (13-25** **°C)**	**Media (RM-MMG)**	**Media (MMG-STR)**	**Temperature (13-25** **°C)**	**Media (RM-MMG)**	**Media (MMG-STR)**
**Total genes differentially expressed (%)** ^**a**^	985 (22)	1037 (24)	1627 (37)	1026 (22)	1362 (29)	2771 (58)
**Total core genes differentially expressed (%)** ^**b**^	763 (21)	809 (22)	1286 (35)	684 (19)	978 (27)	2143 (59)
**Contribution of core genes to total genes differentially expressed**	77%	78%	79%	67%	72%	77%
**Total flexible genes differentially expressed (%)** ^**c**^	222 (29)	230 (30)	342 (45)	342 (30)	384 (34)	629 (56)
**Contribution of flexible genes to total genes differentially expressed**	23%	22%	21%	33%	28%	23%

RM, nutrient rich marine medium.

MMG, minimal medium with 1% glucose.

STR, starvation.

Genes with a log2 fold change of more than 2 are considered differentially expressed.

^a^percentage = value/total number of genes in the genome.

^b^percentage = value/total core genes.

^c^percentage = value/total flexible genes.

The gene content of the flexible genome, in contrast to the core genome, differs widely between AltDE and AltDE1, representing 17 and 24% of their respective genomes, mostly due to the presence of the large plasmid (pAMDE1) in AltDE1. In the transcriptomes, the percentage of the flexible genes in each genome that were differentially expressed was slightly higher than that observed among the shared genes (Table 2). The differential expression within a strain was also very marked (quantitatively) for some flexible genomic regions. For example, the AltDE Integron (fGI-3) between 13 and 25°C or fGI8 between MMG and STR (Figure 1). Using the total genes that are differentially expressed in each condition as a reference, the differentially expressed flexible genes account for 21-33% of that total (Table 2). This is notable since a recent study using the RNA sequencing of *Clostridium difficile* reported a much lower effect (<5%) on the flexible genome when examining differences between growth conditions [24]. Our data clearly show that in addition to a significant contribution from core genome components flexible genes play an important role in the observed changes between conditions and strains.

The transcriptional data of AltDE and AltDE1 also show that of all of the hypothetical proteins in these genomes (~30% in each), the vast majority, 93% (1136 of 1220) in AltDE and 90% (1077 of 1192) in AltDE1, are expressed. In fact, hypothetical genes account for ~50% of the most highly altered genes between two conditions (Additional file 1: Table S2). In AltDE, for example, 56 of the 100 most highly up- or down-regulated genes between 13 and 25°C were annotated as hypothetical, similar to that of AltDE1 (54 of 100). This data highlights the need for further investigation of hypothetical genes, while it also provides some insight into which ones likely play an important role in adapting to variable growth conditions.

### Effects of starvation

Despite the low genome coverage and the low percentage of genes expressed in AltDE1, the difference between AltDE and AltDE1 in the STR condition is notable. The number of reads obtained from the STR transcriptomes was comparable, if not higher, than the other conditions (Table 1), suggesting that additional sequencing would not have necessarily provided significantly higher genome coverage. Despite the lower coverage in these conditions, we detected at least 86% expressed genes in AltDE and 43% in AltDE1 (Table 1). This leads us to speculate that the lower AltDE1 gene expression observed in STR could be the result of a more rapid physiological adaptation rather than a lack of sequencing depth.

Notably, 56% of the total AltDE1 transcriptomic reads under STR mapped to two ncRNAs, tmRNA and RNaseP (discussed below). This contrasts with AltDE, in which only 21% of reads mapped to the same ncRNA. Along with these two ncRNAs, the *csbD* gene was the most highly expressed in AltDE1 during STR (Additional file 1: Table S3). In *Bacillus subtilis* the expression of this gene, mediated by sigma-B, was induced in response to phosphate starvation, although its exact function remains unknown [25]. Other highly expressed genes encoded two ribosome-associated proteins, ribosome modulation factor (RMF) and ribosome release factor (RRF). In *Escherichia coli* RMF was shown to have an influence on the protection against acid stress during stationary phase [26] and heat stress [27] acting as a protective factor of ribosomes during times of slow growth. Moreover, RRF is involved in the dissociation of ribosomes from mRNA and is essential for bacterial growth [28]. Although more evidence is necessary, these data indicate that AltDE1 may undergo a significant transcriptional shift, which alters the ribosomal processes in a way that allows long-term survival during STR and differs from AltDE.

### CRISPR/Cas system and Prophages expression

Transcriptomic data also provides an important tool to assess whether prophages participate in the physiology of these two representatives of *A. macleodii*. Both AltDE and AltDE1 harbor a prophage inserted in their genomes (GI-3 in AltDE and GI-7 in AltDE1), and both appear to encode recognizable structural, replication and recombination proteins (e.g. capsid and tail proteins, helicases, terminases, integrase etc.) [1, 3] Neither of these shows any kind of similarity to known *Alteromonas* phage isolates [29], and it is not known whether or not either of these can enter lytic cycles and produce viable phages. However, in both strains several genes are expressed in these regions (Figure 2), suggesting some activity. In AltDE, the locus showed higher expressions levels in several genes in STR, akin to the CRISPR-locus expression, while only a few genes were expressed in the other conditions. In total, over 37 of the 71 genes were found to increase in expression by at least 2-fold in STR compared to the levels seen in MMG. This data suggest that under this condition the AltDE prophage is apparently induced and could trigger the entry into lytic cycle. In AltDE1, 58 and 47 of the 82 genes of the lambda-like prophage were up-regulated in MMG in relation to RM and STR respectively. These data demonstrate that under adverse conditions, such as starvation, prophages are not passive elements of the bacterial genome but may be active participants in cell physiology. In both prophages there were some genes expressed in all the conditions that change their expression levels in response to environmental perturbations. Other examples of such genes have been reported as cargo genes, which are non-essential for phage proliferation and instead confer adaptive advantages against environmental stress [30] and are potentially involved in virulence [31].

Previously, we reported the presence in AltDE genome of a complete set of CRISPR and CRISPR-associated sequence (Cas) proteins completely absent in AltDE1 [8]. The CRISPR locus has been shown to protect bacteria against foreign nucleic acids such as plasmids and viruses [32]. One of the most significant responses to STR was the increased expression of CRISPR in the AltDE genome (Figure 2) while in the other conditions only minimal expression was detected in RM at 25°C. Interestingly, the entire repeat array was expressed in the STR condition while only a small region was detectably expressed in other conditions. These data suggest that the CRISPR/Cas system may be involved in other cellular functions as has been demonstrated previously for biofilm formation [33] and DNA repair [34].

### Motility

**Figure 4:**
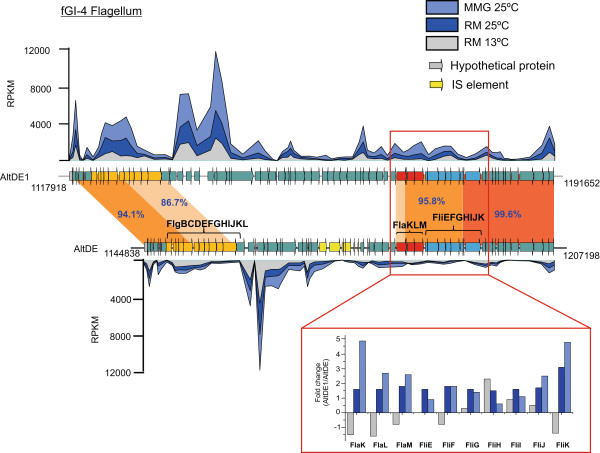
**Expression of the flagellar genes cluster in AltDE and AltDE1.** Gene expression data (expressed as RPKM) is shown mapped to both the clusters. The region enlarged in the red box indicates the expression (fold change) among the regulator genes between both strains in all the conditions (MMG: minimal medium with glucose, RM: Rich Medium).

AltDE and AltDE1 genomes differed with regard to their flagellar operons and that AltDE1 displayed significantly higher motility than AltDE when grown in RM at 25°C [3]. Examination of the gene expression levels of numerous motility related genes revealed that a cluster of genes within the flagellar operon, including the regulatory genes *flaKLM* and the structural genes *fliEFGHIJK*, showed different expression levels in AltDE1 (compared to AltDE) in both RM at 25°C and the MMG (Figure 4). However, at 13°C, this cluster was not up-regulated. Although all the genes of this cluster showed higher expression in AltDE1 only *flaK, flaL flaM, fliJ* and *fliK* demonstrate up-regulation (fold change >2 in AltDE1) during growth in MMG and *fliK* in RM at 25°C. If this cluster of genes is responsible for the difference in motility between the two strains, we would expect differences at 25°C (RM and MMG) but not in RM at 13°C. This was confirmed using motility assays where AltDE1 exhibited significantly greater motility in both RM at 25°C (p < 0.001, paired *T*-test, 2-tailed) and MMG (p < 0.01, paired *T*-test, 2-tailed), while no significant difference was found in RM at 13°C, even though both strains appeared motile, though less than at 25°C (Additional file 1: Table S4). Despite exhibiting an up-regulation in their expression, nucleotide similarity between both genomes was nearly identical in the *fliK* genes (99.6%); therefore, such variability observed could be due to the regulatory genes *flaK* and *flaL,* which sequence were remarkably variable (80 and 84.9%, respectively). The transcriptomic and motility data provide additional support for our previous suggestion that the FlaKLM regulatory system plays a role in the variable motility response of AltDE and AltDE1 to different environmental conditions [3].

### Expression of Non-coding RNA

In addition to examining the differences in gene expression between AltDE and AltDE1, our RNA-seq data allowed us to map the expression of putative non-coding RNA genes (ncRNAs), previously unannotated in *A. macleodii*. ncRNA are small functional RNA that are not translated into a protein, but rather play important roles as post-transcriptional regulators involved in diverse bacterial processes [35]. After excluding putative tRNAs, sixteen transcripts not overlapping to any previously annotated coding region were identified in the AltDE and AltDE1 chromosomes, while none were identified in the plasmid pAMDE1. Fourteen of these, ranging in size from 70 to 359 nt, were identified as putative ncRNA genes using Rfam 10.1 [36] and Bacterial Small Regulatory RNA (BSRD) [37] databases, and two remain unidentified (Table 3). Twelve ncRNAs were found in both strains, and four were found specifically in AltDE1.

**Table 3:** **Expression of ncRNAs in AltDE and AltDE1**

Strain				AltDE1 (RPKM)				AltDE (RPKM)			
ncRNA	Start*	End*	Length (bp)	RM-13°C	RM-25°C	MMG-25°C	STR-25°C	RM-13°C	RM-25°C	MMG-25°C	STR-25°C
TPP riboswitch	152009	152120	111	87.5	35.0	289.9	912.0	100.9	0.0	356.9	461.9
G.II catalytic intron	167749	167819	70	91.0	0.0	114.0	0.0	--	--	--	--
Ribonuclease P	891973	892323	350	39927.4	30365.4	77672.5	925092.0	30718.2	26092.6	98179.9	107875.0
AltRNa1	1066159	1066582	424	20613.4	28361.8	143798.5	90692.6	5133.8	5444.4	61103.3	48553.8
Small SRP	1239467	1239563	96	67.0	80.0	502.0	0.0	63.9	116.4	97.6	0.0
TPP riboswitch	1258919	1259022	103	31.1	0.0	234.1	0.0	123.9	138.8	58.2	235.0
Trp leader	1634441	1634532	91	35.1	42.1	0.0	0.0	0.0	0.0	0.0	0.0
tmRNA	2126827	2127186	359	13072.1	11252.9	56769.8	775951.9	12566.4	10450.9	53778.6	72022.3
His leader	2929451	2929568	117	109.5	0.0	68.8	0.0	6.5	47.5	39.9	80.4
6S RNA	3357865	3358047	182	423.6	529.1	1241.9	558.1	32.9	96.1	161.2	488.1
Thr leader	3471219	3471330	111	64.5	75.2	0.0	0.0	16.1	0.0	0.0	291.4
yybP-ykoY leader	3679956	3680065	109	117.5	70.4	147.6	464.2	--	--	--	--
Alpha RBS	4064601	4064678	77	27744.0	30238.6	5827.2	1964.1	21842.2	19355.7	8201.8	6306.7
AltRNa2	4312594	4312902	309	292.6	1215.7	2206.4	41812.3	949.2	1393.1	1168.1	1047.9
G.II catalytic intron	4420296	4420366	70	45.5	327.3	0.0	0.0	--	--	--	--
G.II catalytic intron D1-D4	4421022	4421188	166	425.5	394.3	291.6	0.0	--	--	--	--

*AltDE1 was used as a reference to the location of the ncRNA. Expression levels are reported here as the normalized RPKM values. RM, nutrient rich marine medium; MMG, minimal medium with 1% glucose; STR, starvation; TPP: thiamine pyrophosphate; SRP: Signal recognition particle; Trp leader: Tryptophan operon leader; tmRNA: Transfer-messenger RNA; His leader: Histidine operon leader; Thr leader: Threonine operon leader; RBS: Ribosome binding site.

Five highly conserved bacterial ncRNAs (Alpha RBS, RNase P, Small SRP, tmRNA and 6S RNA) were found in both strains. Notably, genes coding for tmRNA and RNAse P were among the most highly expressed in all of the transcriptomes, with the greatest levels associated with oligotrophic conditions. In AltDE1, for example, these two ncRNA were the two most highly expressed genes when grown in minimal medium and exhibited increased tmRNA (4X) and RNAse P (2X) levels in MMG compared to their expression in RM at 25°C (Additional file 1: Table S3). RNAseP is a ribozyme that was initially shown to catalyze the removal of the 5' end of pre-tRNAs to produce the mature transfer RNAs (tRNAs) [38]. However, recent discoveries have revealed that is also involved in transcriptional machinery and in the maturation of other RNA substrates, such as mRNAs, precursor tmRNA and riboswitches [39]. tmRNA is a molecule with properties of both a tRNA and an mRNA that is required to release stalled ribosomes and target nascent polypeptides for degradation. Evidence of increased tmRNA levels in cells under different types of stressful conditions [40, 41], including starvation [42, 43] suggests that the cell might increase the tmRNA levels to deal with incompletely-translated and stalled proteins resulting from stress. Ecologically, this is relevant because increased levels of tmRNA observed during starvation likely provide a needed source of amino acids for the synthesis of new proteins from the degradation or recycling of the aberrant proteins [44]. Furthermore, *A. macleodii* is an *r-*strategist (i.e., bloomer) that takes advantage of sporadic inputs of high nutrients in the normally depleted oligotrophic sea, requiring the ability to sense and rapidly respond to nutrient availability. For this purpose, ncRNAs are the perfect regulators due to their immediate availability since they do not require the translation into protein and because of the wide range of cellular responses they can modulate.

We have also found several riboswitches, small RNA that can regulate gene expression by directly binding a small molecule ligand [45]. One of these highly conserved riboswitches was found in the 5′-untranslated region of the thiamine pyrophosphate (TPP) operon and is known to be involved in TPP regulation through the prevention of TPP transcription [46]. RNA leader sequences are another type of riboswitches classified as *cis*-acting ncRNAs. Within the AltDE and AltDE1 chromosome three additional predicted RNA leader sequences involved in amino acid (histidine, tryptophan and threonine) biosynthesis were identified (Additional file 1: Table S2). A third type of *cis*-acting ncRNAs, a Yybp-ykoY leader, was found in the AltDE1 genome only, though the specific function of these ncRNAs is unknown. Along with Yybp-ykoY leader, there were another three ncRNA that were only present in AltDE1. These genes belonged to group II intron RNAs, which are both mobile genetic elements that catalyze their own splicing as well as catalytic RNAs [47].

For the two remaining non-coding transcripts (AltRN1 and AltRN2), no homologies were found in any ncRNA databases. BlastN searches against the NCBI nucleotide database showed that AltRN1 and AltRN2 had matches only to several representatives of *A. macleodii* (AltRN1 > 99% nucleotide identity to all *A. macleodii* strains belonging to the deep ecotype; AltRN2 > 99% and 88-87% identity to deep and surface *A. macleodii* ecotypes, respectively)*.* AltRN1 expression in both strains was comparable to the expression of ribosomal genes, and consistently higher in minimal media. Moreover, AltDE1 expressed this gene at higher levels across conditions (Additional file 1: Table S2). For these reasons, AltRN1 would be a good candidate for further study as a potential new ncRNA.

### Differences in population structure during co-culture

The transcriptional data suggests that AltDE1 may have an increased advantage under circumstances where glucose is abundant. However, from these data alone we cannot discern whether these differences result in any ecological advantage/disadvantages *per se*. To address this issue, we co-cultured AltDE and AltDE1 in RM and MMG in order to determine whether the transcriptional differences observed resulted in a competitive advantage for AltDE1. We also developed a plating assay utilizing differential antibiotic resistance between the two strains to enumerate the colony-forming units (CFUs) from each. The AltDE1 strain carries a large plasmid containing a hybrid non-ribosomal peptide synthetase-polyketide synthase (NRPS-PKS) genes cluster, which is predicted to produce a bleomycin-like compound and contains a bleomycin-resistance gene [23]. We have shown before that AltDE1 is also resistant to Phleomycin (a bleomycin family compound). As a result we could determine CFUs for both strains utilizing marine agar plates with and without Phleomycin. The plates without antibiotic gave us the total count of both AltDE and AltDE1, while the Phleomycin plates represented only the AltDE1 colonies due to its resistance. AltDE colonies were then calculated by subtracting the AltDE1 CFUs from the total CFUs. The CFU results revealed colonies of both AltDE and AltDE1 when the co-culture was grown in RM (Additional file 2: Figure S2A). In contrast, the co-cultures grown in the MMG resulted in the presence of AltDE1 colonies only (Additional file 2: Figure S2A). These data show, as suggested by the transcriptomic data, that AltDE1 increases its advantage over AltDE under specific conditions in which glucose is the sole source of carbon and that this advantage is not simply based on a faster growth rate.

We also designed a specific PCR strategy exploiting the divergent O-chain region between the two strains, which allowed us to determine relative population levels of both strains in all of the conditions examined. The strain specific amplification of the O-chain gene, admittedly only semi-quantitative, provided an initial indication that both strains maintained relatively consistent populations in co-culture for up to two weeks in the RM at both temperatures (Additional file 2: Figure S2B). Although this contrasted slightly with the CFU results, the discrepancy is likely due to the fact that the region of the AltDE1 O-chain amplified is larger than that of AltDE causing some variation in optimal amplification. Regardless, the populations shifted dramatically in the minimal medium condition (i.e., glucose as sole carbon source), where only the AltDE1 O-chain was amplified (Additional file 2: Figure S2C). This corroborated the CFU findings, in accordance with the transcriptomic analysis, that AltDE1 does indeed have an advantage over AltDE when glucose is the sole carbon source. This could be ecologically significant and explain certain strain-level variation as a function of environmental parameters.

## Conclusions

Two closely-related strains of *A. macleodii*, with an ANI of 98.6% and isolated from the same Mediterranean seawater sample, have been shown to harbor significant genomic variation [1, 3, 48]. Here we use RNA-seq to provide evidence that these genetic differences result in variable transcriptomic profiles. Most significantly, the differences between AltDE and AltDE1 resulted in different adaptation to growth in minimal medium and particularly under starvation. This study also provides evidence that these transcriptional differences influence the population dynamics of AltDE/AltDE1 co-cultures under specific conditions. The apparent advantage of AltDE1 when glucose is the sole carbon source disappears in the presence of rich media, suggesting that AltDE displays a higher affinity for carbon sources other than glucose and illustrating the complementary capacities previously proposed to characterize natural bacterial populations [49]. This highlights the concept that the ever increasing diversity observed within closely related groups of microorganisms, often in the flexible genomic islands, most likely plays a vital role in the ability of a microbial community as a complex mixture of different clonal lineages to adapt to environmental shifts.

## Methods

### Growth of *A. macleodii*strains

The two strains of *A. macleodii* used in this study have been fully sequenced and described previously: AltDE [9] and AltDE1 [3]. Both strains were initially grown on marine agar plates (3.5% sea salt, 0.5% peptone, 0.1% yeast extract, and 1.5% agar), with inoculum taken from frozen glycerol stocks and examined for purity prior to use. Individual colonies were grown in the RM, marine media (3.5% sea salt, 0.5% peptone, 0.1% yeast extract), at 25°C overnight, and 4 mL of the AltDE and AltDE1 inoculum were each transferred to 96 mL of MB. The optical density (OD600) was measured using a spectrophotometer (BioSpectrometer®; Eppendorf) and 150 μL of 1.0 OD600 AltDE and AltDE1 were each used to inoculate 15 mL of media for each of 4 growth conditions. The first two conditions were designed to compare differences between different growth temperatures, therefore, AltDE and AltDE1 were grown in the marine medium at either 13 or 25°C until they reached mid-late exponential phase (OD600 = 0.7-0.9). The second set of conditions was designed to examine differences between feasting and starving conditions, thus AltDE and AltDE1 were grown in minimal medium (3.5% sea salt, 0.2% ammonium chloride, 0.05% potassium phosphate, 100 mM Tris–HCl) with or without 1% glucose at 25°C. The “feasting” samples were subsequently collected at mid-late exponential phase (OD600 = 0.7-0.9). The “famine” samples were collected at the same time; however, they were centrifuged at 3000 rpm for 5 min, and the cells were washed with minimal medium and finally resuspended in 15 mL of minimal media without glucose. They were grown for 48 hours additional hours in minimal media without glucose and then collected for RNA extraction. Cultures for all conditions were performed in triplicate.

### RNA isolation

The cultures were centrifuged at 7500 rpm for 15 min, resuspended in RNAlater® Solution (Ambion # AM7024, USA) and used directly for RNA extraction. Before total RNA extraction with the RNeasy® Mini Kit (Qiagen #74106) in accordance with the instruction from the manufacturer, cultures were incubated for 10 min at room temperature in 500 μl of TE (Tris–HCl 10 mM, EDTA 1 mM, pH 8.0) with lysozyme (2 mg/ml) and proteinase K (0.4 mg/ml). Genomic DNA was removed from the extracted RNA by treating the samples with DNAse I (Sigma-Aldrich #AMPD1-1KT) at room temp for 30 min. Agarose gel electrophoresis and staining confirmed the absence of genomic DNA in the RNA.

### cDNA synthesis

Total RNA (10 μg) was used to make single-stranded cDNA using High Capacity cDNA Reverse Transcription (Applied Biosystems #4368814) per the manufacturer’s instructions. The second strand was synthesized by adding 30U of E. coli Polymerase I (New England Biolabs #M0209L), 5U of E. coli DNA Ligase (New England Biolabs #M0205S), 5 U of RNase H (Epicentre #R52250), 300 μM of dNTPs (Invitrogen #18427-013) to the first strand reaction. After 2,5 h at 16°C, 5U of T4 DNA polymerase (New England Biolabs #M0203S) was added to the sample and maintaining at 16°C during 40 min. Finally the double-stranded cDNA was cleaned with a QIAquick PCR Purification kit (Qiagen #28104) and quantified using the ND-1000 Spectrophotometer (NanoDrop, Wilmington, USA). The quantity and quality of all cDNA samples were determined on an Agilent 2100 bioanalyzer.

### Sequencing and transcriptomic data analysis

cDNA from the three biological replicates of each condition described above was pooled and used directly for sequencing using the Illumina HiSeq 2000 (100-bp paired-end read) sequencing platform (GATC Biotech). Sequence reads were pre-processed to remove low-quality bases, and reads shorter than 30 bp. Reads were first mapped using Bowtie software [50] against rDNA operon sequences of AltDE and AltDE1 strains. Remaining reads were subsequently mapped to the AltDE and AltDE1 chromosome sequence with the default parameters. SAMtools [51] were used to convert resulting data into BAM format. BamView [52] was subsequently used for the visualization of the sequence reads against the AltDE and AltDE1 genomes. Once the transcripts were mapped to the genomes, the expression values of genes were calculated as the number of reads aligned over each coding DNA sequence (CDS). Gene expression values were computed using RPKM normalization (reads per kilobase pair of transcript per million mapped reads). Genes exhibiting a >2 fold log change in RPKM between two conditions or strains were considered to have differential expression. To quantify high and low expression values, we used the average median value for all conditions within both strains as a cutoff. Cluster analysis and heat maps were prepared using the Multiexperiment Viewer (MeV) of the TM4 microarray software suite [32]. The transcriptome sequencing data has been submitted to NCBI SRA and is available with the accession numbers SRX447917 - SRX447925 (SRA Bioproject identifier PRJNA236321).

### Co-culture experiments

AltDE and AltDE1 were both grown in marine media (as described above) to an OD600 1.0, at which point 5 μl of AltDE and 5 μl of AltDE1 were each added to 100 mL of the appropriate media in three separate, sterile flasks, providing triplicate biological replication. In addition, 10 μl of AltDE and 10 μl of AltDE1 were added to sterile flasks containing the same media (100 mL) separately to act as controls for each experiment. Co-cultures were performed using each of the four growth conditions described above. Subsamples (2 mL) were taken from the co-cultures and controls after 1, 7 and 14 days of growth. At each time point, DNA was extracted from the resulting cell pellet using QIAprep Spin Miniprep kits (Qiagen 27106) for PCR analysis (described below) and CFUs were counted for the day 7 samples (described below).

### PCR amplification of the strain specific o-chain region

PCR amplification was performed for each DNA sample described above using primers designed to target the O-chain gene cluster of AltDE and AltDE1. This region of the genome has been shown to be quite distinct between the two strains [3], and we were able to design the following primers that target only AltDE or AltDE1: AltDE-ochain-for 5'GATGCCGCCCATTTTGATCC3'; AltDE-ochain-rev 5'AGGTTGGAGACCAAAGCTCG3'; AltDE1-ochain-for 5'AACGATGAAGGGAGCTCGTG3'; and AltDE1-ochain-rev 5'TAGCGCAGTGGCTGAAAGAA3'. All PCR reactions were performed using BIOTAQ DNA polymerase (BioLine BIO-21040) in 50 μL reactions as follows: 37.5 μL sterile water, 5 μL 10× reaction buffer, 4 μL of MgCl2 (25 mg/mL), 1 μL of a dNTP mix (10 mM), 1 μL each primer (10 μM) and 0.5 μL BIOTAQ polymerase (5U/μl). A PTC-100 Peltier Thermal Cycler (MJ Research Inc.) was used with the following temperature cycling profile: 1 cycle at 95°C for 5 min; 23 cycles at 94°C for 45 s, 57°C for 45 s and 72°C for 1 min; and 1 cycle at 72°C for 10 min. The cycle number was optimized using AltDE/AltDE1 DNA in known quantities. All PCR amplification products were visualized using electrophoresis on 0.8% agarose gels with a 1Kb ladder as a size reference.

### Colony Forming Units (CFUs)

In a prior work, we showed that AltDE is susceptible to the antibiotic phleomycin (Invivogen), while AltDE1 is not [23]. We used that distinction to design an assay, which enumerates the CFUs for each strain during co-culture of AltDE and AltDE1 when grown in either RM or MMG. After 7 days of co-culture an aliquot was taken and serial dilutions plated on both marine agar, in which both strains grow, and marine agar with phleomycin (100 ug). The CFUs from the latter plates (representing the number of AltDE1 cells) were then subtracted from the CFUs for the marine agar plates (representing total bacteria cells), providing an estimate of AltDE cells as well. Each experiment included AltDE and AltDE1 pure culture as controls, while the co-cultures were done in triplicate for biological replicates.

### Motility assay

AltDE and AltDE1 were inoculated (0.1%) into 50 ml of each of the following: RM 13°C, RM 25°C, and MMG 25°C. They were grown to an OD600 1.0, and 1 μL of each culture was stabbed into the center of a 0.25% RM or MMG agar plates. The plates were then incubated at the appropriate temperature for 48 h, and the diameter of the growth zone was measured at 24 and 48 h. Each experiment was performed in triplicate for both strains, and statistical analyses were performed on the combined data from 24 and 48 hours using the three data sets for each condition. We used a two-tailed student’s *t*-test to determine whether there was significant (p < 0.05) variation between AltDE and AltDE1 motility.

## Electronic supplementary material

Additional file 1: Table S1: Expression levels of nitrogen-associated genes. **Table S2.** The number of hypothetical genes differentially expressed in AltDE and AltDE1. **Table S3.** AltDE and AltDE1 genes differentially expressed in response to starvation conditions. **Table S4.** Results of motility assays for AltDE and AltDE1. (XLSX 27 KB)

Additional file 2: Figure S1: Expression of 300-kb conjugative plasmid (pAMDE1) in the AltDE1 genome. Gene expression data is expressed as fold change among the conditions. Grey region show the location of the NRPSPKS cluster. (MMG: minimal medium with glucose,STR: starvation, minimal media without glucose, RM: Rich Medium). **Figure S2.** Differences in population structure during co-culture. A) Colony Forming Units (CFUs) reveal significant differences in the number of AltDE and AltDE1 cells during co-culture. P-values were calculated using two-tailed, paired t-tests with statistical significance determined for pvalues < 0.05. B) Strain-specific PCR amplification targeting the variable O-chain region of both strains (AltDE – 390bp, AltDE1 – 700bp) also reveals a shift in the population structure of AltDE and AltDE1 grown in co-culture between RM and MMG. (PDF 2 MB)
