# Supplementary material for: RNA sequencing provides evidence for functional variability between naturally co-existing Alteromonas macleodii lineages
Source: BMC Genomics. 2014 Oct 26;15(1):938. doi: 10.1186/1471-2164-15-938 (PMC4223743; doi:10.1186/1471-2164-15-938)
Supplement: Supplementary file 2 — Additional file 2: Figure S1: Expression of 300-kb conjugative plasmid (pAMDE1) in the AltDE1 genome. Gene expression data is expressed as fold change among the conditions. Grey region show the location of the NRPSPKS cluster. (MMG: minimal medium with glucose,STR: starvation, minimal media without glucose, RM: Rich Medium). Figure S2. Differences in population structure during co-culture. A) Colony Forming Units (CFUs) reveal significant differences in the number of AltDE and AltDE1 cells during co-culture. P-values were calculated using two-tailed, paired t-tests with statistical significance determined for pvalues < 0.05. B) Strain-specific PCR amplification targeting the variable O-chain region of both strains (AltDE – 390bp, AltDE1 – 700bp) also reveals a shift in the population structure of AltDE and AltDE1 grown in co-culture between RM and MMG. (PDF 2 MB) [file 12864_2014_6629_MOESM2_ESM.pdf]

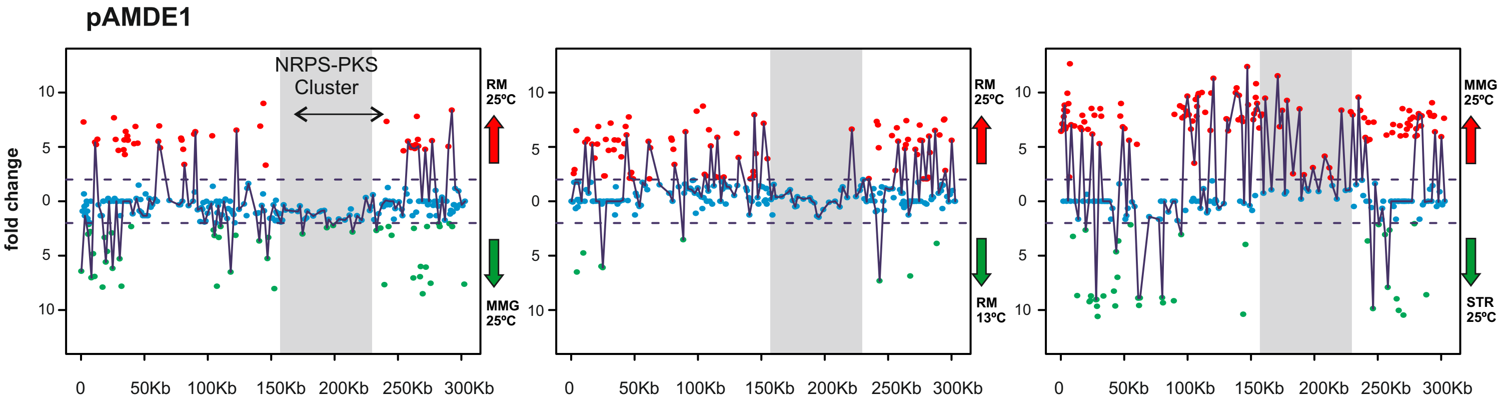

Supplementary Figure 1. Expression of 300-kb conjugative plasmid (pAMDE1) in the AltDE1 genome. Gene expression data is expressed as fold change among the conditions. Grey region show the location of the NRPS-PKS cluster. (MMG: minimal medium with glucose,STR: starvation, minimal media without glucose, RM: Rich Medium).

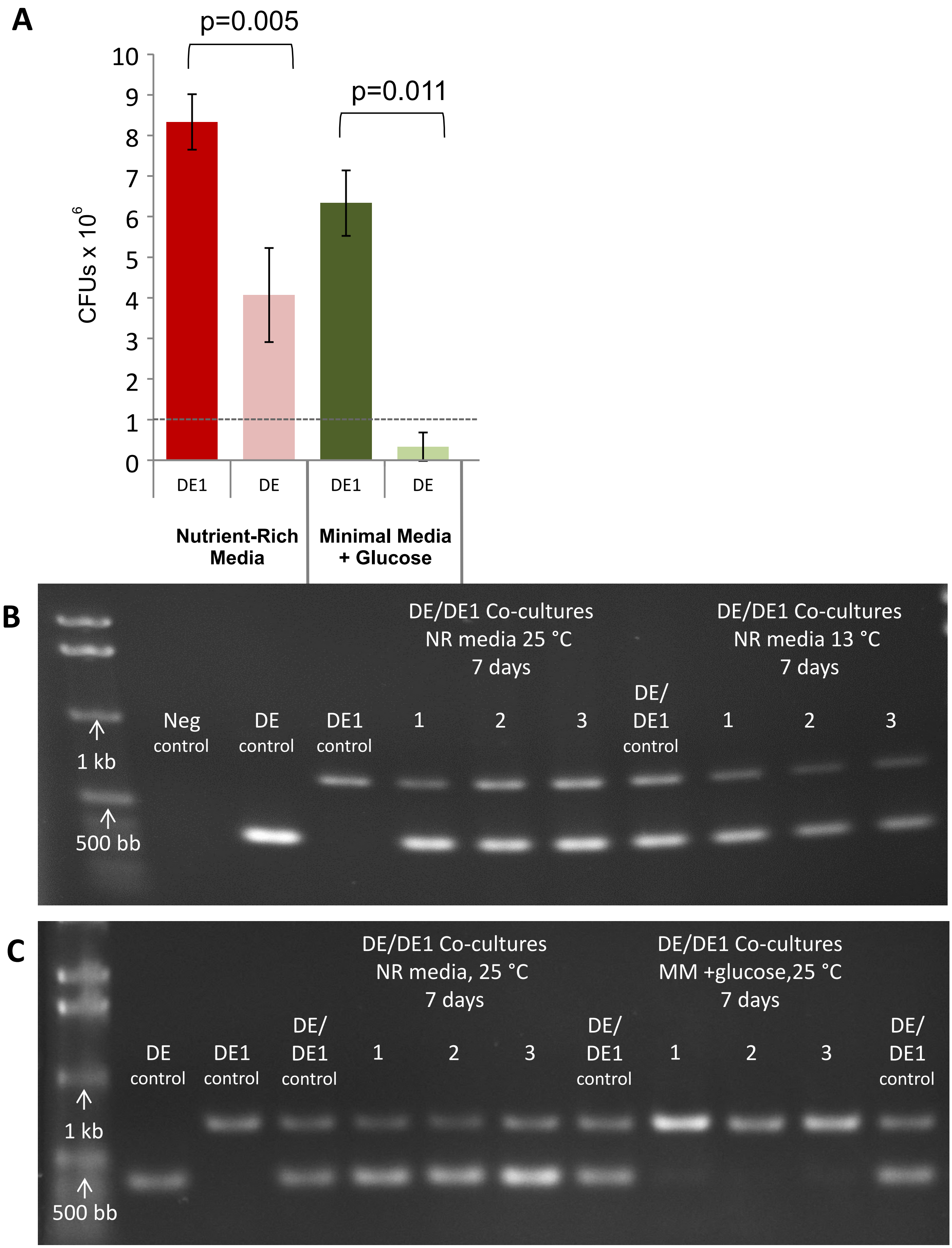

Supplementary Figure 2. Differences in population structure during co-culture. A) Colony Forming Units (CFUs) reveal significant differences in the number of AltDE and AltDE1 cells during co-culture. P-values were calculated using two-tailed, paired t-tests with statistical significance determined for pvalues < 0.05. B) Strain-specific PCR amplification targeting the variable O-chain region of both strains (AltDE – 390bp, AltDE1 – 700bp) also reveals a shift in the population structure of AltDE and AltDE1 grown in co-culture between RM and MMG
